# Causal Interactions between Frontal^θ^ – Parieto-Occipital^α2^ Predict Performance on a Mental Arithmetic Task

**DOI:** 10.3389/fnhum.2016.00454

**Published:** 2016-09-14

**Authors:** Stavros I. Dimitriadis, Yu Sun, Nitish V. Thakor, Anastasios Bezerianos

**Affiliations:** ^1^Institute of Psychological Medicine and Clinical Neurosciences, Cardiff University School of MedicineCardiff, UK; ^2^Cardiff University Brain Research Imaging Center, School of Psychology, Cardiff UniversityCardiff, UK; ^3^Artificial Intelligence and Information Analysis Laboratory, Department of Informatics, Aristotle University of ThessalonikiThessaloniki, Greece; ^4^Neuroinformatics.Group, Department of Informatics, Aristotle University of ThessalonikiThessaloniki, Greece; ^5^Singapore Institute for Neurotechnology, Centre for Life Sciences, National University of SingaporeSingapore, Singapore

**Keywords:** working memory, cognition, cross-frequency coupling, mental arithmetic, hierarchical organization

## Abstract

Many neuroimaging studies have demonstrated the different functional contributions of spatially distinct brain areas to working memory (WM) subsystems in cognitive tasks that demand both local information processing and interregional coordination. In WM cognitive task paradigms employing electroencephalography (EEG), brain rhythms such as θ and α have been linked to specific functional roles over given brain areas, but their functional coupling has not been extensively studied. Here we analyzed an arithmetic task with five cognitive workload levels (CWLs) and demonstrated functional/effective coupling between the two WM subsystems: the central executive located over frontal (F) brain areas that oscillates on the dominant θ rhythm (Frontal^θ^/F^θ^) and the storage buffer located over parieto-occipital (PO) brain areas that operates on the α_2_ dominant brain rhythm (Parieto-Occipital^α2^/PO^α2^). We focused on important differences between and within WM subsystems in relation to behavioral performance. A repertoire of brain connectivity estimators was employed to elucidate the distinct roles of amplitude, phase within and between frequencies, and the hierarchical role of functionally specialized brain areas related to the task. Specifically, for each CWL, we conducted a) a conventional signal power analysis within both frequency bands at F^θ^ and PO^α2^, b) the intra- and inter-frequency phase interactions between F^θ^ and PO^α2^, and c) their causal phase and amplitude relationship. We found no significant statistical difference of signal power or phase interactions between correct and wrong answers. Interestingly, the study of causal interactions between F^θ^ and PO^α2^ revealed frontal brain region(s) as the leader, while the strength differentiated between correct and wrong responses in every CWL with absolute accuracy. Additionally, zero time-lag between bilateral F^θ^ and right PO^a2^ could serve as an indicator of mental calculation failure. Overall, our study highlights the significant role of coordinated activity between F^θ^ and PO^α2^ via their causal interactions and the timing for arithmetic performance.

## Introduction

Based on a psychology theory, the human working memory (WM) system possesses a star topology with the executive element in the center and supportive elements in the periphery. The central executive is the core of the human WM system and controls and organizes information selection and processing. Peripheral WM subsystems store task-relevant information for the short term and can be called buffers (e.g., like a visuospatial sketch of a visual stimulus).

Neuroimaging studies have identified the brain regions involved in accessing these storage systems, providing new anatomic insight for better understanding the coordination of neural activities from multiple brain regions in the WM system (Sauseng et al., 2005; Kawasaki et al., 2010; Zanto et al., 2011). It was previously assumed that the prefrontal cortex (PFC) affects processing in posterior brain regions (Friese et al., 2013; Szczepanski et al., 2014; Harding et al., 2015). This assumption led to the hypothesis that PFC activity should precede parietal activity in cognitive control (Brass et al., 2005). It is generally accepted that top-down signals from frontal areas are important for cognitive control (Desimone and Duncan, 1995; Miller and Cohen, 2001; Corbetta and Shulman, 2002). Frontal activity, specifically in the PFC, is thought to affect posterior regions and facilitate the processing of task-relevant information (Reynolds and Chelazzi, 2004; Maunsell and Treue, 2006). Two time series {X,Y} [e.g., electroencephalography (EEG) oscillations] have a causal relationship when past values of X can be useful for predicting future values of Y. This terminology of causality was first formulated by Granger (1969). The above assumption is supported by the time precedence of prefrontal activity (Brass et al., 2005; Grent-’t-Jong and Woldorff, 2007), synchronization between prefrontal and posterior regions (Buchel and Friston, 1997; Sakai and Passingham, 2003), and modulation of posterior region activity after inactivation of prefrontal regions via transcranial magnetic stimulation (TMS; Taylor et al., 2007).

The causal role of the PFC modulating evoked activity in the extrastriate cortex during experiments related to scenes and faces (Miller et al., 2011) and recent combined TMS/EEG and TMS/ functional magnetic resonance imaging (fMRI) studies (Feredoes et al., 2011; Higo et al., 2011; Zanto et al., 2011) clearly supports the idea that the PFC is the source of top-down signals and plays a key role in allocating attentional resources related to semantic long-term memory (e.g., letters, numbers, sounds, and motor or sensory information; D’Esposito and Postle, 2015).

Neural activity and related oscillations can be studied at many levels using spike trains, local field potentials (LFPs), and large-scale oscillatory activity that can be measured with EEG. For large-scale oscillations, amplitude changes due to variable synchronization in a neural ensemble are usually referred to in the literature as local synchronization. Oscillatory activities between distant neural structures (neural ensembles or single neurons) can also be synchronized. Neural synchronization and oscillations with a specific frequency profile have been associated with various cognitive functions like memory, perception, motor control, and information transfer (Fries, 2005; Fell and Axmacher, 2011).

EEG oscillations are thought to reflect the orchestration of cell assemblies via the synchronization of neurons related to specific functions and the activities of many local cell assemblies linked to different functions that are integrated via large-scale synchronization (Varela et al., 2001). This hypothesis is the basis for the use of EEG-dominant oscillations for exploring dynamic functional connectivity (Dimitriadis et al., 2012). Numerous EEG studies using scalp recordings have demonstrated modulated θ and α brain rhythms in anatomically restricted brain regions and simultaneous phase synchronization between them during various WM tasks (Jensen and Tesche, 2002; Mizuhara et al., 2004; Sauseng et al., 2005; Kawasaki and Watanabe, 2007; Klimesch et al., 2008; Kawasaki et al., 2010). Each EEG oscillation is closely related to the functional role of each anatomically distinct brain area linked to one or more separate cognitive functions (Başar et al., 2014). Even though there is a debate about the link between cognitive functions and the dominant interaction type, there is ample evidence that cross-frequency coupling (CFC) based on phase domain may be the substrate that links these frequency-independent cognitive functions (e.g., WM subsystems; Fell and Axmacher, 2011). For instance, Kawasaki et al. (2010) suggested that WM task-relevant brain regions [i.e., Frontal (F) and Parieto-Occipital (PO) regions] are coordinated by an *m*:*n* phase synchronization between θ oscillation (θ, 5–8 Hz) in frontal (F^θ^) and α_2_ oscillation (α_2_, 10–13 Hz) at PO regions (PO^α2^). In a systematic review, Fell and Axmacher (2011) highlighted that phase–amplitude coupling and *m*:*n* phase coupling are crucial for a non-interfering representation of multiple objects in the WM.

Among various WM tasks, mental arithmetic is often used to investigate the neurophysiologic basis of WM. Two studies based on EEG event-related responses for mental subtraction and addition adopted a calculation strategy that highlighted the importance of θ and α oscillations (De Smedt et al., 2009; Grabner and De Smedt, 2011). Moreover, evidence from several neuroimaging studies indicates that several cortical areas distributed over both hemispheres are generally implicated in arithmetic processing (Menon et al., 2000; Gruber et al., 2001; Dehaene et al., 2003, 2004; Kong et al., 2005; Fehr et al., 2007; Ischebeck et al., 2009). For example, multiplication operations demand the retrieval of arithmetic facts (e.g., multiplication tables) that are stored in the verbal memory (manipulation of verbal numbers) and specifically require left angular gyrus activation (Gruber et al., 2001; Dehaene et al., 2003; Ischebeck et al., 2009). In contrast, subtraction and addition demand a calculation where the two numerical quantities must first be represented as quantities, and this process activates regions in the parietal cortex (Chochon et al., 1999; Menon et al., 2000; Dehaene et al., 2003; Fehr et al., 2007). It is important to understand the different roles of each brain area involved in mental arithmetic (Dehaene et al., 2004), their preferred “working” frequencies (Ishii et al., 2014), and possible cross-frequency coordination to obtain a correct outcome (Roux and Uhlhaas, 2014).

In our first attempt, we aimed to differentiate two extreme cognitive workload levels (CWLs): the addition of single-digit numbers (CWL-1) with three-digit numbers (CWL-5) based on a single-subject dataset. We successfully separated the two CWLs using highly accurate connectivity patterns (Dimitriadis et al., 2013). Based on these promising results, we focused on how to correctly predict any CWL among the five CWLs. We discriminated on a high recognition-rate (96%) the five CWLs by constructing the first functional connectivity graph (FCG) that incorporates intra-frequency phase coupling within frontal and PO brain areas operating on their dominant oscillation (θ and α, respectively) and the inter-frequency phase-to-phase coupling between those two areas. The FCG was analyzed with a tensorial approach (Dimitriadis et al., 2015b). As the next step, we attempted to predict performance in arithmetic calculations based on the previous analysis and a causal relationship between prefrontal and parietal activity in which cognitive control was addressed (Brass et al., 2005). To study causal relations between F^θ^ and PO^α2^, we introduced a new information-theoretic method based on symbolic transfer entropy that quantifies the strength, direction, and delay of coupling between neural signal activities in different frequency ranges (Dimitriadis et al., 2015d). We found that this method successfully uncovering the leading role of θ band oscillation in F areas over α_2_ frequency oscillation in PO at each CWL (Dimitriadis et al., 2016a).

**FIGURE 1:**
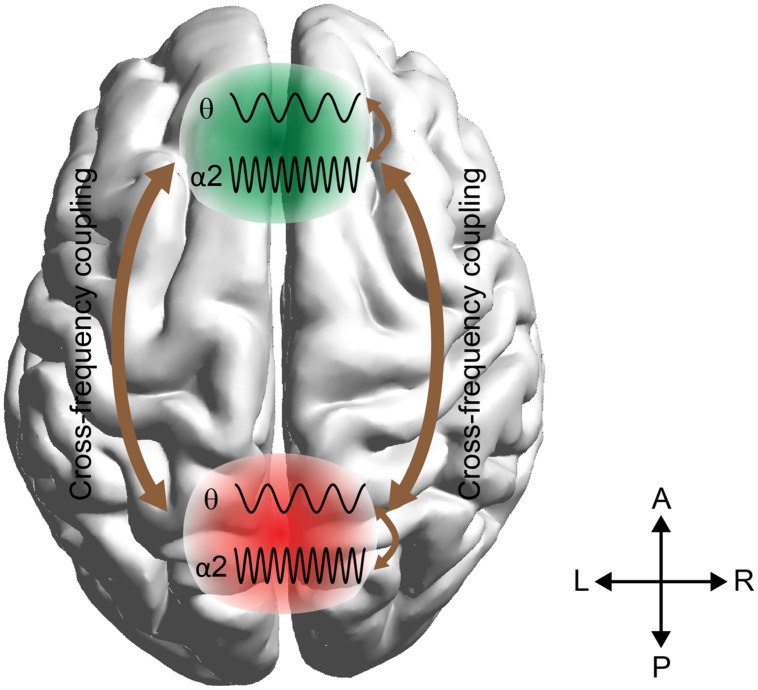
**Schematic illustrations of working memory (WM) manipulation of the visual representations.** The external visual information is stored in the modality-specific posterior region by the α2 rhythm. For manipulation of the stored representations, the cross-frequency phase coupling (CFPC) between θ–α2 connect the frontal (F) executive regions with parieto-occipital (PO) regions where the visual and number related information is stored. Moreover, θ–α2 interact within both F and PO regions.

Here we employ these techniques to predict arithmetic performance. The scope of this work was fourfold: (i) to uncover the different roles of amplitude and phase representation of θ and α_2_ activity while performing mental calculations, (ii) to study the different types of phase synchronization between F^θ^ and PO WM subsystems, (iii) to address and quantify the causal interactions between the two WM subsystems (F^θ^ and PO^α2^), and (iv) to investigate how WM subsystems can be coordinated for coherent cognitive function. To address these questions, we analyzed the correct and wrong answers in an EEG arithmetic task (addition) with varying difficulty (Lv; i.e., increasing the numbers of digits in the added numbers). Our hypothesis was based on the model in **Figure 1** where stimulus-related information is stored in the posterior brain areas via the α_2_ brain rhythm, while manipulation of the abstract-stored information is manipulated by the frontal executive brain areas under θ rhythm via the cross-frequency coupling pathway (Kane and Engle, 2002).

## Materials and Methods

### Subjects

We recruited 16 young, right-handed volunteers (nine males and seven females, ages 21–26 years, mean age of 21.5 [*SD* = 1.5 years]) from the National University of Singapore. All participants had normal or corrected-to-normal vision and reported that they did not have verbal or non-verbal learning disabilities. The study was approved by the Institutional Review Board of the National University of Singapore conforming with the Code of Ethics of the World Medical Association (Declaration of Helsinki), and written inform consent was obtained from each participant after the procedures were clearly explained.

### EEG Recordings

EEG data were recorded from 64 channels at 256 Hz with an ActiveTwo Biosemi system and referenced using an average reference. The experiment details were described previously (Dimitriadis et al., 2015b, 2016a). Briefly, we asked each participant to perform the mental summation of two numbers presented on a PC screen. When the subject finished the arithmetic task, he/she pressed the spacebar, and two possible answers appeared on the screen. The subject had to compare the mentally calculated summation with the two options. The participant was asked to press either the left or right arrow key (⇐ or ⇒) that corresponded to the correct answer. If the participant responded correctly or incorrectly, the trial was characterized as correct or wrong, respectively. The experiment design included five difficulty levels that differed by the number of digits of the added numbers. For the first level, arithmetic problems involved the summation of two one-digit numbers (e.g., 7 + 9). For each subsequent level, the number of digits of one of the numbers increased by one; therefore, at level five the mental arithmetic task consisted of the summation of two three-digit numbers (e.g., 235 + 164). The experimental paradigm was divided into 1-min with 30-sec rests to avoid cognitive fatigue. All the arithmetic problems within each block had the same difficulty level. After a three repetitions of the five difficulty levels, subjects relaxed by viewing a slide show with landscape pictures with a frequency of 1 picture every 30 sec. We selected landscapes over a fixation cross to increase subject alertness. Then, the whole session was repeated with 15 blocks of mental arithmetic and 5 min of relaxation. Due to the different response times, each subject performed a different number of trials for each block.

A single trial was defined as the time interval between stimulus onset and the last peak of θ (6 Hz) cycle^1^ before the subject pressed the space bar.

### Data Preprocessing

We performed independent component analysis (ICA; Onton et al., 2006; Delorme et al., 2007; Romero et al., 2008) to suppress artifactual activity, after first concatenating the trials using the EEGLAB package (Delorme and Makeig, 2004). Independent components (ICs) marked as artifacts (eyes, muscle, and cardiac interference) were zeroed (Dimitriadis et al., 2010). Afterward, we reconstructed the multichannel signal from the non-artifactual ICs using the estimated mixing matrix.

### Determining Frequency Bands and Recording Sites of Interest

Previous works (Sauseng et al., 2005; Klimesch et al., 2008; Kawasaki et al., 2010) revealed a distinct role of frequency bands originating from brain areas related to specific cognitive roles in WM and mental arithmetic tasks. Based on this knowledge, we targeted our connectivity analysis to selected brain areas with a characteristic frequency profile. In multiplication and comparison tasks, an increment of θ power over frontal brain areas in both hemispheres and a decrement of power in α_2_ frequency over PO sites in both hemispheres were detected for both tasks (Micheloyannis et al., 2005). First, based on preliminary analysis published in our previous study (Dimitriadis et al., 2015b), we demonstrated that higher cognitive loads are linked to: (i) increased power in θ (5–6 Hz) and α_2_ (10–13 Hz) frequency bands over F brain areas bilaterally and (ii) increased power in θ and α_2_ over PO regions bilaterally (for power spectrum (PS) estimation see **Supplementary Material Section 1**). Sensors located over bilateral F and PO that demonstrated this tendency in θ (5–6 Hz) and α_2_ (10–13 Hz) power, respectively, were selected for further analysis. The selected sensors included FZ, FP1, AF3, F3, F7, FC5, FC1, FC6, FC2, F4, F8, FP2, AF4, PZ, P7, P8, P5, P6, PO7, PO8, PO3, O1, OZ, O2, and PO4.

### Different Types of Connectivity Estimators

To uncover the different roles of amplitude and phase on predicting task performance and determine their potential causal relationship within and between different frequencies, we adopted a repertoire of established connectivity estimators and a novel one (Dimitriadis et al., 2016a). Specifically, we employed the phase locking value (PLV; Lachaux et al., 1999), directed phase lag index (dPLI; Stam and van Straaten, 2012), phase-to-amplitude cross-frequency coupling (PAC; Cohen, 2008; Voytek et al., 2010; Dimitriadis et al., 2015a, 2016b; Adamos et al., 2016), and delay symbolic transfer entropy (dSTE; Dimitriadis et al., 2016a).

#### Within and Cross-Frequency Phase Synchronization between F^θ^ and PO^α2^

To quantify the phase interaction within F^θ^ and PO^α2^ and also between the two brain areas functioning on their prominent frequency, we adopted *PLV* as an index to quantify phase synchronization on a single-trial basis. The center frequency for θ was 6 Hz, and the range for α_2_ was 10–13 Hz (for further details see **Supplementary Material Section 2**). Briefly, *PLV*s were estimated in a pairwise fashion from the brain signals recorded at sensors *k*, *l* oscillating on prominent frequencies (i.e., θ for F areas and α_2_ for PO areas) with the following formula:

$$PLV\left(x_{k}(f,n),x_{l}\left(f,n\right)\right)=|\frac{1}{\left(W.\Delta s\right)}\overset{W}{\underset{n^{\prime }=1}{\Sigma }}\overset{s_{2}}{\underset{s=s_{1}}{\Sigma }}\exp\left(i\left(\varphi^{x_{k}}(f,n^{\prime },s)-\varphi^{x_{l}}\left(f,n^{\prime },s\right)\right)\right)|$$

Here, *x*_*k*_*(n)* denotes a single trial (correct or wrong response) segment of length equal to *N*_w_ samples extracted from addition of one of the CWLs. Φ_*k*_(*s*_1_,*n*) defines the instantaneous phase for the single-trial segment over N_s1_ scales within either the θ or α_2_ frequency bands. W equals the variable width in samples of each trial. Phase estimation via Morlet wavelet transform with a Gaussian envelope in the time domain (characterized by standard deviation σ_t_) produced a complex number located at a center frequency f with resolution σ_f_ ranging from σ_1_ to σ_2_ (Tallon-Baudry et al., 2001). Then, single-trial phase synchronization was quantified with the *PLV* formula between preselected EEG sensors over F and PO brain sites within θ or α_2_ frequency bands (i.e., *PLV*(^θ^*x_k_*, ^θ^*x_l_*) and *PLV*(^α2^*x_k_*, ^α2^*x_l_*)).

We also estimated the phase-phase cross-frequency coupling between F^θ^ and PO^α2^ between every pair of sensors from F and PO brain areas as a coordinated mechanism between the two functionally distinct WM subsystems (i.e., central executive and storage buffer). Adopting the concept of *n*:*m* phase synchronization (Tass et al., 1998), we modified Eq. 1 to estimate the cross-frequency phase-to-phase differences between two cycles of θ phases (2 × Φ_6Hz_) and one cycle of the α_2_ phase (Φ_10-13Hz_) between the preselected sensors over F and PO brain areas:

PLV(xk(fθ,n),xl(fα2,n))=|1(W.Δs)Σn′=1WΣs=s1s2exp⁡(i(2*φ6Hz(fθ,n′,s)−φ10−13Hzxk(fα2,n′,s)xl))|

*PLV* (*^fθ^x_k_*, ^fα2^*x_l_*) quantified the information exchange rate between distinct WM subsystems (for further details – see **Supplementary Material Section 2**). We previously found that cross-frequency coupling significantly improved the classification performance of CWLs for correct trials (Dimitriadis et al., 2015b).

To assess the statistical significance level of *PLV* estimates, we used the Rayleigh test for the uniformity of *PLV* values as previously described (Dimitriadis et al., 2013, 2015a,b,c,d, 2016a,b; for further details see **Supplementary Material Section 2B**).

#### Effective Connectivity and Time Delay between F^θ^ and PO^α2^

##### Dynamic causal modeling, structural equation modeling, and granger causality

Effective connectivity is defined as “the influence one system exerts over another” (Friston, 2011). It is important to mention that functional connectivity is not necessarily effective connectivity. Only effective connections are directed from one brain area to another. Many techniques have been proposed to explain causal interactions in multichannel neuroimaging recordings, including like dynamic causal modeling (DCM), structural equation modeling (SEM), and Granger causality (GC).

GC is a statistical concept of causality that predicts the future of an activity X based on the past activity Y. Two time series {X,Y} (e.g., EEG/MEG/fMRI oscillations) have a causal relationship when past values of X can be useful for predicting future values of Y. This terminology of causality was first formulated by Granger (1969). In the last 10 years, GC has gained much attention in the neuroscience community (Kaminski et al., 2001; Barnett and Seth, 2014).

The main goal of DCM in neuroimaging data based on a set of regions of interest (ROIs) is to determine the causal influence of each ROI on the others and study the experimental influence on the connections’ strengths. The aforementioned procedure demands: (i) biophysically and physiologically plausible models of neuronal network dynamics that can predict the pattern of the network topology that better described the connectivity of a spatially distinct predefined subset of ROIs related to the experimental stimuli and (ii) efficient and computationally feasible statistical parametric estimations and model comparisons that better fit the experimental data, leading to meaningful directed networks that are supported by the literature (Friston et al., 2003). DCM is a Bayesian model comparison procedure between candidate models that better describes experimental data. Dynamic causal models are described via ordinary differential equations that are non-linear state-space models. These equation-based models describe the dynamics of hidden states in a set of nodes of a probabilistic graphical model where their conditional dependencies are parameterized via directed effective connectivity. The probabilistic graphs in DCM can be cyclic (the estimated graphs cannot be cyclic in GC or SEM), and DCM does not assume that random fluctuations are uncorrelated (Downey and Hirschfeldt, 2010), unlike GC and SEM.

The aim of DCM is to infer the causal architecture of coupled or distributed dynamical systems. This Bayesian model comparison procedure rests on comparing models of how data were generated. Dynamic causal models are formulated in terms of stochastic or ordinary differential equations (i.e., non-linear state-space models in continuous time). These equations model the dynamics of hidden states in the nodes of a probabilistic graphical model, where conditional dependencies are parameterized in terms of directed effective connectivity. Unlike Bayesian networks, the graphs used in DCM can be cyclic, and unlike SEM and GCM, DCM does not depend on the theory of algorithmic randomness (Downey and Hirschfeldt, 2010).

In fMRI, DCMs typically rely on two classes of states, namely “neuronal” and “hemodynamic” states. The latter encodes neurovascular coupling for modeling fMRI signal variance generated by neural activity (Friston et al., 2003). Biophysical models in DCMs for EEG/magnetoencephalography (MEG)/LFP data are typically more complex than in DCMs for fMRI. This is because the richness in temporal information contained by electrophysiologically measured neuronal activity can only be recorded by neurobiological models. The report introducing DCM for EEG/MEG data (David et al., 2006) relied on a so-called “neural mass” model.

Following the initial paper by David et al. (2006), a number of extensions to this “neural mass model”-based DCM were proposed that considered both spatial and temporal aspects of MEG/EEG data. Concerning the spatial domain, one problem is that the position and extent of cortical sources are difficult to precisely specify *a priori*. Kiebel et al. (2006) proposed to estimate the positions and orientations φ of “equivalent current dipoles.” Concerning the temporal domain, computational problems can arise when dealing with recordings of enduring brain responses where it is more efficient to summarize the measured time series in terms of their spectral profile. This is the approach developed by Moran et al. (2007, 2008, 2009), which models LFP data based on the neural mass model using a linearization of the evolution function f around its steady state (Moran et al., 2007).

Structural equation modeling analyses start with a set of ROIs and try to estimate the connection strengths between the predefined ROIs leading to a model that best describes the connectivity pattern. The connections in the model are directional and represent the degree of correlation between the time series describing ROI activities. SEM procedures can vary, but most of them are based on general linear modeling (GLM) searching through the space of possible sets of connections that best fit the data (Graham, 2008). A comparison between dynamic (DCM) and static (SEM) effective connectivity analyses was published by Penny et al. (2004a,b).

For a better understanding of the various techniques of DCM, SEM and GC, an interested reader should refer to the original papers, the main extensions of the techniques and to well-presented reviews (Daunizeau et al., 2011; Friston, 2011; McIntosh and Mišić, 2013).

##### Causal interactions and time delay between F^θ^ and PO^α2^

To unfold both causal interactions and the time delay between F^θ^ and PO^α2^, we adopted the dSTE method (Dimitriadis et al., 2016a). Compared to GC (Granger, 1969), the adopted technique of STE is a model-free approach of effective connectivity that is not affected by outliers or filtering and can be directly applied to non-stationary multichannel recordings. This is in comparison to GC that assumes only stationary signals and is affected by various filtering options (for a comparison study see Papana et al., 2013). Partial direct coherence (PDC) and direct transfer function (DTF) are the best options when we analyze time series from linear systems that operate on the same frequency. In our case, we proposed for the first time a causality estimator called dSTE for time series with different frequencies that can detect the strength, direction and lag between non-linear and non-stationary multichannel recordings (Dimitriadis et al., 2016a; see **Supplementary Material Section 3**).

To strengthen our hypothesis that activity in frontal brain regions precedes parietal activity in cognitive control (Brass et al., 2005), we adopted a new technique that addresses the time lag between two time-series based on cross-correlation of instantaneous amplitudes of two oscillations (Adhikari et al., 2010) (see **Supplementary Material Section 4**). The technique was adopted supplementary to the time-lag detection based on our target method (see **Supplementary Material Section 3**).

#### Uncovering Causal Phase Relationship between F^θ^ and PO^α2^

We detected and quantified the strength of causal phase interactions between F^θ^ and PO^α2^ brain areas via the dPLI (Stam and van Straaten, 2012). The dPLI obtains the phase difference between two time series (A and B) based on the following formula:

$$dPLI=\frac{1}{N}\overset{N}{\underset{t=1}{\Sigma }}H\left(f\left(t\right)\right)$$

There are three cases based upon the distribution of the phase difference within [*-π, π*]:

(a) Most of the phase differences between two time series are in the interval of 0 ≤ Φ(t) ≤ π, and signal A is consistently leading in phase domain signal B with a *dPLI* > 0.5
(b) Phase difference of the two signal are on average π radians out of phase where we cannot say anything about who drives who
(c) Most of the phase differences between two time series are in the interval of π ≤ Φ(t) < 0, and signal B is consistently leading in phase domain signal A with a *dPLI* < 0.5

After applying the proper statistical filtering approach (see **Supplementary Material Section 5**), we aggregated the strength of the significant causal relationship between F^θ^ and PO^α2^ brain sites.

#### PAC between F^θ^ and PO^α2^

Cross-frequency interactions between F^θ^ and PO^α2^ were also assessed via PAC. The algorithm was adopted to our EEG multichannel recordings as described below. Let *x*(*i*, *t*) be the EEG activity recorded at the *i*th site, *x*(*j*, *t*) be the EEG activity recorded at the *j*th site, and *t* = 1, 2, …, T, represents the successive time points. Given two frequency band-limited signals *x*(*i*, *t*) and *x*(*j*, *t*), cross-frequency coupling and namely PAC directly estimated the strength of the phase of the low-frequency (LF) oscillations to modulate high-frequency (HF) oscillation amplitude. The complex analytic representations of both signals *Z_LF_*(*t*) and *Z_HF_*(*t*) are extracted via the Hilbert transfer (HT):

$$\begin{array}{c} Z_{LF}(t)=HT[X_{LF}\left(t\right)]=Z_{LF}e^{i\Phi LF\left(t\right)}=A_{LF}\left(t\right)e^{i\Phi LF\left(t\right)}\\ Z_{HF}(t)=HT[X_{HF}\left(t\right)]=Z_{HF}e^{i\Phi HF\left(t\right)}=A_{HF}{\left(t\right)}^{i\Phi HF\left(t\right)} \end{array}$$

Then, the envelope of the HF oscillations, *A_HF_*(*t*) is filtered within the range of LF oscillations (here in θ) and from the filtered signal, phase dynamics φ’(t) are derived via an HT:

$$Z^{\prime }(t)=HT\left[A_{HF,LF}\left(t\right)]=|Z^{\prime }(t)\right|e^{i{\Phi^{\prime }}_{{HF}^{\left(t\right)}}}=Z^{\prime }{\left(t\right)}^{i\Phi_{LF\to HF\left(t\right)}}$$

The aforementioned formula describes the modulation of the amplitude of HF-oscillations by the phase of LF-oscillations and adopting *PLV* (Eq. 1) as an index of the PAC strength, we can quantify the phase consistency of these two time series. Again, we aggregated the strength of significant PAC estimates between F^θ^ and PO^α2^ (Dimitriadis et al., 2015a, 2016a; see **Supplementary Material Section 6**).

### Accessing Classification Performance between Correct and Wrong Trials

We commence this subsection by including the classification strategy using a set of features including signal characteristics, behavioral data, PS, phase coupling, and dSTE. The total number of wrong trials within the group was 277 and distributed in each CWL as followed: *Lv1* = 42, *Lv2* = 53, *Lv3* = 59, *Lv4* = 62, and *Lv5* = 61 (Lv = level of difficulty). Since the individual total number of wrong trials across CWL ranged from 1 to 13, we trained the binary classifier by manipulating correct and wrong trials from the whole group. The above procedure will strengthen our results by presenting the classification performance with a group-unified classifier. The classification scheme was based on PS, phase coupling within F and PO brain areas on their prominent frequency (θ in F and α_2_ in PO), cross-frequency phase-to-phase coupling between F^θ^ and PO^α2^, PAC between the phase of F^θ^ and the amplitude of PO^α2^, directed cross-frequency amplitude-to-amplitude coupling based on dPLI between F^θ^ and PO^α2^, and dSTE between F^θ^ and PO^α2^. This scheme was followed for each CWL independently between correct and wrong trials (binary classification).

For PS and reaction time measurements, we employed the Laplacian score as a feature extraction technique (He et al., 2005), and a k-NN classifier served as the predictor of correct versus wrong trials for each CWL. In the feature-extraction step, signal-power was separately estimated from the selected EEG sensors for each single-trial segment for θ band P^θ^, α_2_ band P^α2^, and the corresponding ratio P^θ^/P^α2^ in both PO and F brain areas. We augmented this large number of features with the reaction time. We further normalized all features within the [0, 1] range. Complementary to PS and reaction times, we classified correct versus wrong trials based on the different representations of FCGs estimated with a large repertoire of connectivity estimators. For the classification strategy, we adopted a previously published approach based on the tensorial treatment of FCGs (Dimitriadis et al., 2013, 2015d,c; Antonakakis et al., 2016). We adopted a 10-fold cross-validation scheme for both signal-power/reaction time and different functional connectivity representations (see **Supplementary Material Section 7**).

## Results

### Behavioral Results

**FIGURE 2:**
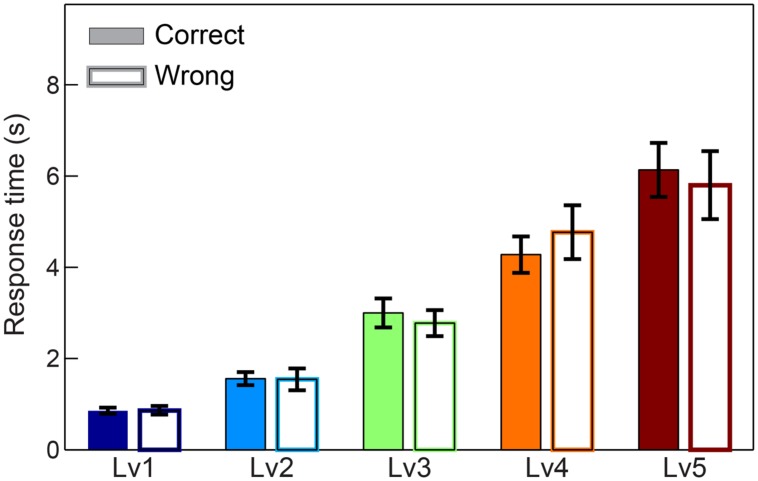
**Response times across five CWL in both correct and wrong trials.** Bars represent mean ± standard error of mean (SEM). No significant difference was observed in the response time between correct and wrong trails.

We first compared response times and subject performance between consecutive CWLs. Adopting Wilcoxon rank-sum tests and Bonferroni corrections, we revealed the expected effect of difficulty for response time (*p* < 0.001, Bonferroni corrected, *p′* < p/4) with longer responses to be linked to higher CWLs and higher difficulty. In terms of accuracy, we demonstrated a decreasing trend across the increment of difficulty, but it was not significant after correcting for multiple comparisons (for *p* < 0.001, Bonferroni corrected, *p′* < p/4) (see **Supplementary Material Section 8**). Additionally, no statistical differences were detected between correct and wrong trials based on response times (**Figure 2**).

### Power Spectrum Evidence in F^θ^ and PO^α2^ Related with Arithmetic Performance

**FIGURE 3:**
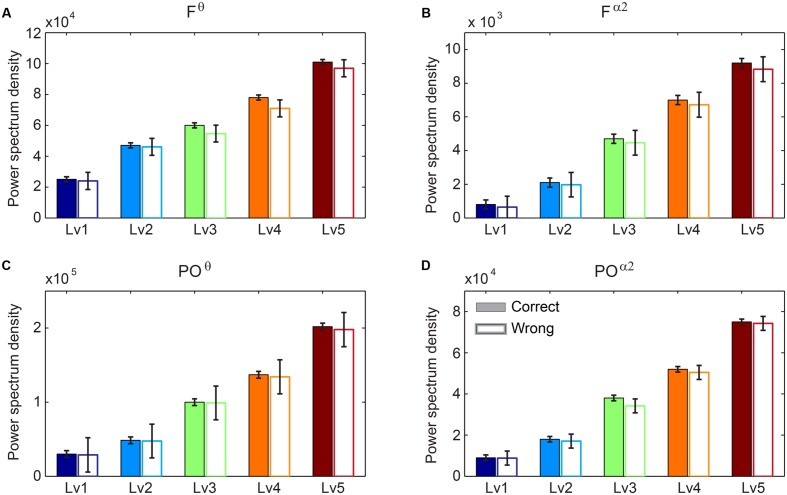
**Group-averaged power spectrum in θ band (5–6 Hz) **(A)** over frontal (F) bilaterally areas and **(C)** over parieto-occipital (PO) sites and in α2 band (10–13 Hz) **(B)** over F bilaterally areas and **(D)** over PO sites for the five CWLs in both correct and wrong trials.** Bars represent mean ± standard error of mean (SEM). No statistical significant differences were detected based on Wilcoxon rank sum test (*p* < 0.001; Bonferroni corrected across CWLs).

We performed Wilcoxon rank-sum tests to find significant differences between correct and wrong responses in terms of the PS (see **Supplementary Material Section 1**). Our analysis did not reveal any significant tendency of the PS in F^θ^ and PO^α2^ regarding the behavioral response (correct *vs.* wrong trials). Interestingly, we found significant increasing trends for both F^θ^ and PO^α2^ following task difficulty in both correct and wrong trials (**Figure 3**).

### Intra- and Inter-frequency Phase Coupling within and between WM Subsystems

**FIGURE 4:**
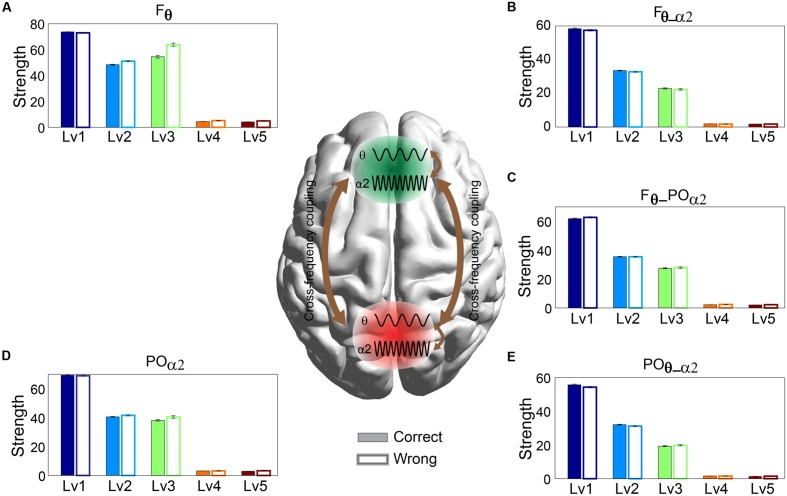
**Strength distribution of within- and between-phase interactions located over F brain regions: **(A)** within-phase interaction in Frontal (F^θ^), **(B)** between-phase interaction in Frontal (F^θ-α2^), **(D)** within-phase interaction in Parieto-Occipital (PO^α2^), and **(E)** between-phase interaction in Parieto-Occipital (PO^θ-α2^) and **(C)** cross-frequency coupling between them (F^2 × θ^ – PO^α2^) for both correct and wrong answers.** Bars represent mean ± standard deviations. No statistical significant differences were detected between correct and wrong trials.

Any significant difference between correct and wrong responses in terms of strength related to different types of phase interactions was analyzed via Wilcoxon rank-sum testing. The subgraph strength for either F or PO brain regions was the total weight of all the connections within each of the brain areas and was estimated for each subject by averaging across trials and for each CWL. We found that the five different types of phase memory synchronization showed a decrement tendency with the increment of the CWL in both correct and wrong trials. However, our analysis failed to reveal any significant difference between correct and wrong responses. Phase interactions within F^θ^ (**Figure 4A**) and PO^α2^ (**Figure 4D**), *n*:*m* (2:1) θ-α_2_ phase interactions between F^θ^ and PO^α2^ (**Figure 4C**), within F^θ-α2^ (**Figure 4B**), and within PO^θ-α2^ brain regions (**Figure 4E**) did not show any significant effect related to arithmetic performance (**Figure 4**).

### Strength of Phase Causal Interactions and Phase-to-Amplitude Interactions

**FIGURE 5:**
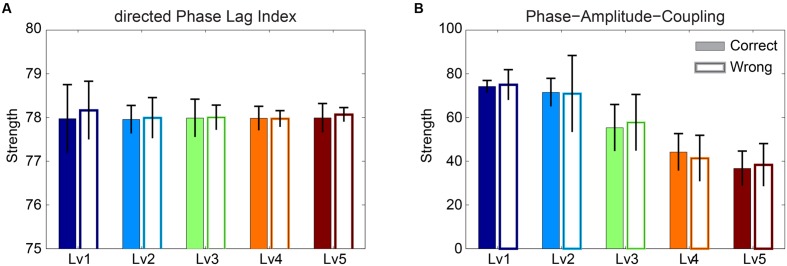
**Strength of **(A)** dPLI and **(B)** PAC between Frontal^θ^ and Parieto-Occipital^α2^ (F^2 × θ^ – PO^α2^) for both correct and wrong answers.** Bars represent mean ± standard deviations. No statistical significant differences were detected between correct and wrong trials.

The significance level of the strength related to dPLI and PAC was assessed with Wilcoxon rank-sum testing. Our analysis failed to reveal any significant difference between correct and wrong responses for both types of brain synchronization (**Figure 5**). Additionally, dPLI did not demonstrate any effect of task difficulty, whereas PAC strength diminished with greater difficulty for both correct and wrong trials.

### Causal Interactions and Time-Lag between WM Subsystems

**FIGURE 6:**
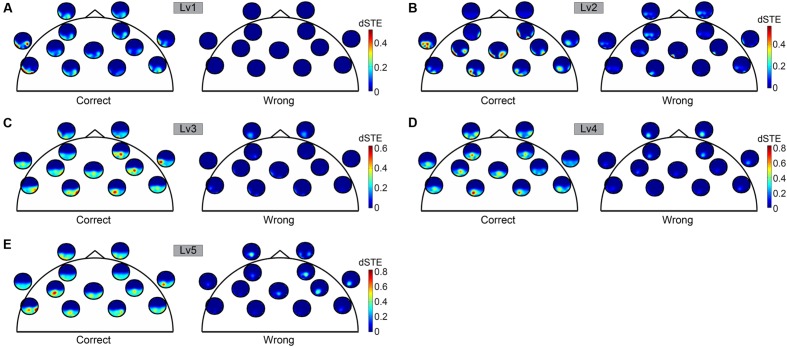
**To represent dSTE for each of the five cognitive workload levels [**(A)** Lv1, **(B)** Lv2, **(C)** Lv3, **(D)** Lv4, and **(E)** Lv5] and in both correct (left panel) and wrong trials (right panel), we adopted a novel visualization scheme, in which the relative position of a single sensor u is used to embed a whole brain’s topography that represents the dSTE(u,v) measurements related to it.** A minute topography depicts the dSTEs from a particular sensor to all other destinations (sensor locations). It serves as a natural display of a row in the dSTE matrix. The topographic representation illustrates only the Frontal sensors because ΔdSTE uncovered F^θ^ brain region as a leader.

**FIGURE 7:**
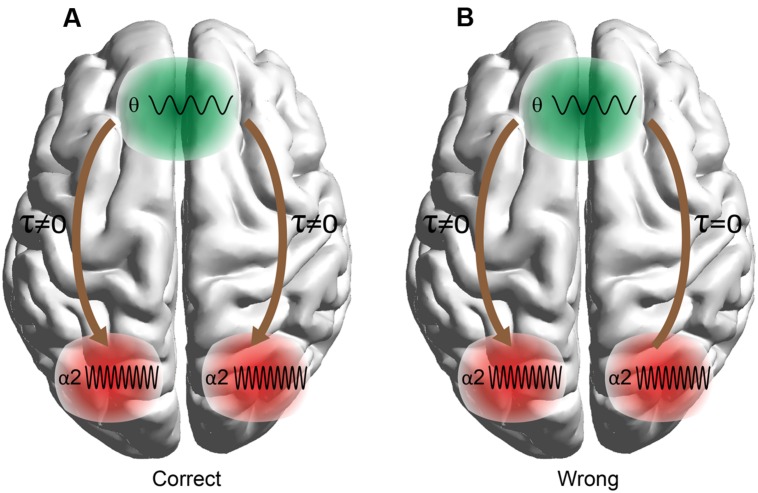
**Time-lag effects between F and PO sites in **(A)** correct and **(B)** wrong trials.** The analysis detected time-lag effects for three out of four combinations (right and left PO × correct and wrong trials) with the exception of bilateral F and right Parieto-Occipital sites in wrong trials.

Before applying statistical tests to causal interactions between correct and wrong answers, we adopted a two-step averaging procedure: (i) averaging across trials for each subject independently and (ii) averaging across subjects. Our statistical analysis was based on Wilcoxon rank-sum tests (*p* < 0.001). To correct for multiple testing, we adopted the false discovery rate (FDR) method to correct for multiple testing and applied it to each trial (Benjamini and Hochberg, 1995). Adopting dSTE to estimate causal interactions between F^θ^ and PO^α2^, we found a significant trend: F^θ^ drives PO^α2^ (**Figure 6**). The statistical analysis uncovered F^θ^-PO^α2^ dysfunction in wrong trials to each CWL compared to correct trials (**Figure 6**). **Figure 6** shows the significant interactions. Both dSTE and cross-correlation of amplitude envelopes between F^θ^ and PO^α2^ show that F activity precedes that of PO in both correct and wrong trials. Importantly, time-lag estimation with both dSTE and cross-correlation of amplitude envelopes revealed an interesting trend in wrong trials compared to correct: right PO (rPO^α2^) brain region (i.e., channels P6, P8, PO8, PO4, and O2) showed zero time-lag with bilateral F brain regions (bF^θ^) across the five CWLs (**Figure 7**). **Table 1** summarizes the group-average time-lag (in ms) between bF^θ^ and rPO^α^_2_ estimated with dSTE^NG^ for each CWL based on correct trials.

**Table 1:** Group-average time-lag (mean ± SD) between bF^θ^ and rPO^α2^ estimated with dSTE^NG^ for each cognitive workload level (CWL) based on correct trials.

Workload level	Lv1	Lv2	Lv3	Lv4	Lv5
Time-lag (ms)	117 ± 14	121 ± 17	114 ± 21	132 ± 14	133 ± 17


### Classification Performance based on Behavioral, Power Spectrum, and Connectivity Analysis

The classification performance, based on PS expressed via a group-averaged correct recognition rate, was 58.8 ± 11.2% (**Table 2**). The Laplacian score only detected the PS as a useful feature to improve the binary classification of correct versus wrong trials at each CWL (see **Supplementary Material Section 7**). The classification performance based on PS was marginally at by chance classification performance (50%). We present the results based on functional connectivity estimates following alternative strategies with the goal of differentiating correct from wrong trials for each CWL using the single-trial subgraph connectivity estimates from all subjects. The group-averaged performance of the tensor subspace analysis + k-nearest neighbor classifier (TSA + k-NN) scheme based on F^θ^, PO^α2^, and the CFC was 65.0 ± 12.1% (**Table 2**). When we applied the TSA + k-NN strategy to the F^θ^ and PO^α2^ subgraphs, the classification performances were 61.1 ± 8.4% for θ and 56.5 ± 8.1% for α_2_, (**Table 2**) (for further details see **Supplementary Material Section 7**). We further analyzed correct and wrong trials based on cross-frequency phase-to-phase coupling between F^θ^ and PO^α^_2_ where the maximum classification performance was held on for the fifth CWL reaching 65.1 ± 12.1%. Phase-to-amplitude coupling between F^θ^ and PO^α^_2_ improved the classification accuracy of phase-to-phase coupling, but this was not significant and reached 68.35 ± 9.3% in the fifth CWL. Cross-frequency coupling was also analyzed via the directionality of the amplitude-to-amplitude activity in F^θ^ and PO^α^_2_ using dPLI and the novel dSTE using the tensorial approach for comparable purposes with the previous connectivity estimators. Based on the strength, effective connectivity graphs that incorporated dPLI showed the highest score for the fifth CWL at 69.4 ± 13.5%. In contrast, effective connectivity graphs that incorporated the strength of dSTE between F^θ^ and PO^α^_2_ (**Figure 7**) revealed a group-averaged performance of the TSA + k-NN scheme equals to 100% (**Table 2**). Additionally, we previously stated that rPO^α2^ showed a zero time-lag with bF^θ^ across the five CWLs in the wrong but not correct trials (**Figure 7**).

**Table 2:** Classification behavioral performance based on power spectrum (PS), phase coupling (PC), phase-to-amplitude cross-frequency coupling (CFC), causal phase relationships directed phase lag index (dPLI) and delay symbolic transfer entropy (dSTE).

	Lv1	Lv2	Lv3	Lv4	Lv5
PS	57.1 ± 13.2%	53.1 ± 10.2%	56.2 ± 9.2%	58.8 ± 11.2%	58.1 ± 9.8%
PC^θ^	55.6 ± 10.1%	56.2 ± 8.7%	61.1 ± 8.4%	54.3 ± 7.8%	57.3 ± 10.2%
PC^α2^	54.3 ± 8.2%	56.3 ± 12.3	55.3 ± 6.5%	56.5 ± 8.1%	53.4 ± 7.3%
PC^CFC^	58.4 ± 7.4%	59.3 ± 9.1%	58.9 ± 12.3%	60.1 ± 10.1%	65.1 ± 12.1%
PAC^CFC^	60.2 ± 9.2%	61.7 ± 8.7%	64.4 ± 10.7%	63.3 ± 9.4%	68.4 ± 9.3%
dPLI^CFC^	61.8 ± 10.1%	65.2 ± 11.3%	65.7 ± 9.5%	65.8 ± 12.5%	69.4 ± 13.5%
dSTE	100%	100%	100%	100%	100%


## Discussion

Two recent dual recording studies (EEG and LFP) uncovered the different roles of amplitude and phase in brain activity (Ng et al., 2013; Musall et al., 2014). Many neuroimaging studies have demonstrated the different functional roles of WM subsystems in various cognitive tasks. EEG WM rhythms such as θ and α are located in the well-defined F and PO brain regions, respectively (Kawasaki et al., 2010). A WM study based on modeling demonstrated that input information is stored in posterior brain areas by α rhythm (Chik, 2013), while short-term-stored representations are manipulated within the frontal executive WM systems by the θ rhythm, which also connects frontal and posterior brain sites (Kane and Engle, 2002). In the present study, we attempted to demonstrate how the central executive and storage buffer WM subsystems operate during an arithmetic task and also how their coordination affects arithmetic performance. We assessed the functionality of both WM subsystems oscillating in the preferred phase with PS analysis, intra-/inter-frequency phase coupling (Palva et al., 2005; Kawasaki et al., 2010; Palva and Palva, 2012; Dimitriadis et al., 2015b,a), and causal interactions between different frequencies on both amplitude (Dimitriadis et al., 2016a) and phase (Stam and van Straaten, 2012).

The current study aimed to shed light on the mechanisms and EEG features that contribute to correct behavioral performance. To validate our analysis, we used PS and different types of connectivity estimators based on amplitude, phase, and their causal interactions between F and PO brain areas. Apart from identifying the best feature that can predict behavioral performance in the mental arithmetic task, we wanted to highlight the potential to link performance in a difficult cognitive task with an individual’s brain activity. Our approach could be used as a framework for alternative complex tasks using either EEG or MEG imaging methods. Additionally, the entire methodology can be employed to design an appropriate intervention for dyscalculic subjects who face problems with numeric calculations by focusing on specific attributes of brain dynamics.

Our main results can be summarized in the following four summary points:

• With regard to the level of oscillatory amplitudes in both WM brain regions, the current study did not identify significant differences between correct and wrong responses in terms the PS estimated on both θ and α_2_ frequencies over F and PO brain regions as an integrated estimator. Additionally, reaction times did not reach a statistically significant level between correct and wrong trials.
• Our connectivity analysis focused on various interaction types within and between the WM subsystems oscillating on the prominent frequencies. We adopted intra- and inter-frequency estimators to uncover causal effects between the different frequencies extracting both the time-lag and coupling strength. The adopted estimators were amplitude and phase-oriented as follows: *n*:*m* phase locking estimator (inter-frequency coupling based on phase), dPLI (inter-frequency coupling based on amplitude), PAC (inter-frequency estimator between the phases and amplitudes of two different frequencies), and *PLV* (intra-frequency coupling) failed to discriminate correct from wrong trials for each CWL.
• By employing dSTE to estimate causal interactions (both strength and direction) between F^θ^ and PO^α^_2_, we successfully classified 100% of correct from wrong trials for each subject independently.
• Both dSTE and cross-correlation of amplitude envelopes revealed that F^θ^ drives PO^α2^ in both correct and wrong trials with the only exception of bilateral F (bF^θ^) and right PO (rPO^α^_2_) sites in wrong trials (**Figure 7**).

### Frontal Midline θ Implication to Mental Arithmetic

Generators of frontal midline θ are located on the dorsal part of the anterior cingulate cortex (ACC) and the adjacent medial PFC; both structures are related to focused attention and many cognitive functions associated with mental calculation (Ishihara and Yoshi, 1972; Sasaki et al., 1996; Asada et al., 1999; Enriquez-Geppert et al., 2014). The ACC encompasses various emotional, cognitive, executive, and visuospatial functions (Bush et al., 2000; Womelsdorf et al., 2010). A recent study based on continuous subtraction noted a significant increment of θ power within the ACC (Ishii et al., 2014). Additionally, the dorsal ACC is part of the distributed attentional brain network (Bush et al., 2000). Our results clearly demonstrate a statistically significant increment of power in F^θ^ over task difficulty but no statistically significant difference between correct and wrong trials (**Figure 3**).

### WM and Mental Tasks

WM tasks like mental calculations require distinct functions such as a storage buffer, central executive functions, and coordination between them. The posterior brain regions play a general role in maintaining actual contents of representations (Todd and Marois, 2004; Vogel and Machizawa, 2004). Previous studies also revealed an attentional control role for the PFC; it is responsible for determining which information will be maintained and updated within the WM but is not involved in the maintenance of the relevant information (Cohen et al., 1997; Smith and Jonides, 1999; Rowe et al., 2000; Kawasaki et al., 2006). Other neuroimaging studies hypothesized that WM processes are controlled via top-down signals from the PFC to the posterior brain areas where the mental representation of the related information is stored (Baddeley, 1992; Miller and Cohen, 2001; Curtis and D’Esposito, 2003). A typical mental arithmetic study involved a task-activated distributed network of brain areas including the frontal cortex and bilateral parietal lobes (Dehaene et al., 2004; Pinel et al., 2004).

WM and selective attention have been extensively viewed as separate cognitive domains. In contrast, a growing number of theoretical assessments and reviews in the fields of neuroscience and psychology have reported that these two domains share commonalities (Awh et al., 2006; Postle, 2006; Chun, 2011). Research primate and human neuroimaging studies have already shed light on the significant role of top-down signaling to enhance brain activity in areas related to the stimuli modality while simultaneously suppressing brain activity for distracted stimuli to the targeted goals. Specifically in visual areas, changes related to excitation levels reflected a simultaneous competitive substrate for items represented in receptive fields and a synchronized pattern of neural ensembles (Reynolds and Chelazzi, 2004). The neuroscience community supports the hypothesis that top-down modulation of external sensory information relies on distant interactions (e.g., between the PFC and parietal cortex) and not an intrinsic functionality of modality-specific sensory cortices (Curtis and D’Esposito, 2003; Gazzaley and D’Esposito, 2007). In a delayed recognition task, Zanto et al. (2011) demonstrated that Frontal-Parietal α band (7–14 Hz) phase coherence served as the substrate for long-distance, top-down modulation and provided clear evidence that top-down modulation mediated by the PFC causally links early attentional processes and subsequent memory performance.

### Prominent WM Frequencies of Frontal and Parietal Brain Regions

It is well known that posterior brain areas maintain information related to the modality while the frontal cortex actively manipulates associated information (Postle et al., 1999; Smith and Jonides, 1999; Curtis and D’Esposito, 2003; Wager and Smith, 2003). An increment of θ activity within frontal brain areas is an indicator for increased cognitive demand and focused attention (Yamada, 1998) while decrement of upper α is an index of distinct functions related to task processing (Klimesch, 1999). Even though our experimental paradigm was not designed with a clear discrimination of the manipulation and retention periods, various studies have established the roles of both frequencies and WM brain areas in mnemonic processes (Micheloyannis et al., 2005; Palva et al., 2005; Fell and Axmacher, 2011; Palva and Palva, 2012). Our results based on signal power and brain connectivity with both intra- and inter-frequency phase-to-phase coupling further support the distinct role of both oscillations θ for manipulation and α_2_ for maintenance, as dominant frequency rhythms in WM subsystems in F and PO, respectively. Additionally, the retention period of the adopted experimental paradigm apart from representation maintenance support other distinct functions like preparation and task-related rule maintenance. Previous EEG studies demonstrated phase synchronization between θ and α_2_ brain rhythms in many brain areas in a high number of WM tasks (Jensen and Tesche, 2002; Mizuhara et al., 2004; Sauseng et al., 2005; Kawasaki and Watanabe, 2007; Klimesch et al., 2008).

### Interplay between Frontal and Parietal Brain Regions

Although many neuroimaging studies suggest that PFC plays a key role in the cognitive control, interplay between the PFC and parietal cortex was emphasized (Brass et al., 2005). This raises a fundamental question about the different contributions of these brain areas in cognitive control. It was assumed that the PFC biases processing in posterior brain regions (Miller and Cohen, 2001; Brass et al., 2005). This assumption led to the hypothesis that neural activity in the PFC should precede parietal activity in cognitive control (Brass et al., 2005). Our study uncovered a causality effect between F^θ^ and PO^α2^ brain regions in the five CWLs (**Figure 6**). The PFC serves to add bias signals to other brain structures (e.g., the parietal cortex) to guide stimulus and response processing toward the desired behavior (Miller and Cohen, 2001; Brass et al., 2005). Additionally, a zero time-lag between rPO^a2^ and bF^θ^ brain regions was detected in wrong trials and across the five CWLs (**Figure 7**). A possible explanation of the above significant observation could be attributed to the arithmetic nature of the task and particularly to right posterior parietal cortex oscillation in the preferred α_2_ frequency in which both frequency and brain region are integrated for semantic understanding (Sauseng et al., 2005; Klimesch et al., 2008) and spatial representation of numbers (e.g., a mental number line Hubbard et al., 2005, 2009; Gobel et al., 2006).

### Involvement of Parietal Brain Areas in Math Calculations

Based on findings from previous studies, the intraparietal sulcus is a systematically activated brain area during number manipulation independently of their notation (i.e., digits, dots, plurals of nouns; Carreiras et al., 2015). For this reason, the intraparietal sulcus is activated in all arithmetic tasks as the neural substrate for manipulating quantities or numbers (Dehaene et al., 2003, 2004; Kong et al., 2005). Both subtraction and addition elicit higher intraparietal sulcus activation compared to other arithmetic tasks like multiplication and division. Compared to multiplication where part of the results are stored in rote verbal memory, the addition of numbers is not learned by rote and demands quantity manipulation (Dehaene et al., 2003, 2004). Inferior and postero-superior parietal lobules have been linked to both counting and subtraction (Dehaene et al., 2003). Compared to the intraparietal sulcus that is mainly activated by number representations, the postero-superior parietal area plays a key role in numerous visuospatial tasks that demand attention with or without WM activation (Mitchell and Cusack, 2008; Olson and Berryhill, 2009). The aforementioned findings suggest that postero-superior parietal cortex activation is related to processing attended stimuli. For that reason, the well-known mental number line, a quasi-spatial representation of numbers organized on their proximity, can be the core semantic abstract representation of numerical quantities. It was clearly demonstrated that the process followed in covert attention that is activated to select target locations in space can also be engaged when numbers in arithmetic tasks are manipulated independently of the notation over the mental number line located in the parietal cortex (Dehaene et al., 2004). The PSP lobule is involved in this spatial-attention hypothesis in both visuospatial tasks where the information is presented and in non-visual arithmetic tasks (Ishii et al., 2014).

### Untangling Arithmetic Performance via the Causal Relationship

To summarize, our findings based on causal interactions of the θ and α_2_ rhythms demonstrate the need for communication between frontal and parietal WM subsystems for better mental arithmetic paradigm outcomes (Fell and Axmacher, 2011). The strength of the causality between F^θ^ and PO^α2^ in correct trials and the zero time-lag between bF^θ^ and rPO^α2^ in wrong trials explain arithmetic performance in the five CWLs and unmask the executive role of the PFC in cognition (Miller and Cohen, 2001; Brass et al., 2005). Additionally, the zero time-lag between rPO^α2^ and bF^θ^ in wrong trials improve understanding of the hierarchical spatiotemporal functionality of brain rhythms underlying cognition (Furl et al., 2013). A scalp-EEG brain connectivity study that employed a WM task revealed the need for connectivity between F^θ^ and PO^α^_2_ activity, supporting the need of hierarchical control between F and PO in many spatiotemporal domains and for various cognitive processes like WM (Kawasaki et al., 2010). Finally, in primates, the PFC exerts executive control over cognition by transmitting signals to parietal brain regions in a rule-based spatial categorization task (Crowe et al., 2013). Studies of lesion subjects revealed clear involvement of the right parietal cortex in mental arithmetic processing. Dehaene and Cohen (1997) presented the first report based on two acalculic subjects with structural lesions affecting the right parietal cortex or left subcortical areas. They demonstrated that the subject with a left structural lesion had impaired rote arithmetic facts that were analyzed based on *a priori* knowledge of numerical quantities. However, the patient with a right inferior parietal lesion exhibited significant impairment of quantitative numerical knowledge, which was more severe for subtraction tasks (Dehaene and Cohen, 1997). In addition, a recent study of cortical electrostimulation in patients with brain tumors confirmed an anatomico-functional organization for arithmetic processing within the right parietal cortex (Della Puppa et al., 2013).

In general, neuronal oscillations at different frequencies were recently connected with basic higher cognitive processes, further supporting the distinct functional role of each brain rhythm during WM [for a review, see (Roux and Uhlhaas, 2014)]. The α brain rhythm expresses the level of inhibition of task-irrelevant activity, while θ rhythm supports the temporal organization of abstract items in the WM (e.g., the intermediate results from the addition tasks in the present study). Pairs of cross-frequency couplings like α-γ and θ-γ have a distinct role in managing WM information [for a review, see (Roux and Uhlhaas, 2014].

Top-down manipulation of processing of sensory information is supported by distant interactions like between the PFC and parietal cortex (Curtis and D’Esposito, 2003; Miller and D’Esposito, 2005; Gazzaley and D’Esposito, 2007) and on interactions within these two subnetworks. Zanto et al. (2011) demonstrated that Frontal-Parietal α band (7–14 Hz) phase-coherence is the substrate for distant top-down modulation via the PFC to other activated distributed brain areas and links attentional processes and related memory performance. Summarizing existing evidence in terms of the distinct role of each frequency in WM tasks, the causal role of the PFC over parietal brain areas, right parietal involvement in quantitative numerical knowledge, and our findings, we can untangle the significant prediction of arithmetic performance. The loss of top-down control of the PFC over the right PO area can be interpreted as an interruption between these two brain areas and the major role of the PFC in overall cognitive control (Brass et al., 2005). A possible explanation of the above significant observation is the arithmetic nature of the task; particularly, the right posterior parietal cortex oscillates in the preferred α_2_ frequency, so both frequency and brain region are integrated for semantic understanding (Sauseng et al., 2005; Klimesch et al., 2008), spatial number representation (similar to a mental number line; Hubbard et al., 2005, 2009), and the loss of inhibition of task-irrelevant activity.

Top-down modulation supports our ability to pay attention to task-relevant stimuli and suppress irrelevant distracted information. The common prediction of arithmetic performance across subjects and in the five CWLs was the inability of the subject to inhibit irrelevant distracted information; they then lost the ability to give a correct answer even on the first levels with simple addition. The current findings are among the first cognitive neuroscience results that adopted a large repertoire of connectivity estimators and succeeded in clarifying the distinct role of each frequency distributed over specific anatomical brain areas, each with a different role in WM arithmetic mental tasks. Finally, our findings will be useful for studying mental arithmetic tasks in aging adults and dyscalculic children, as well as guiding neurofeedback strategies.

### Limitations

One of the major limitations of the present study is that the sensor space connectivity analysis lacks the higher spatial resolution that would be achieved by performing the same analysis in the source space. Despite tremendous improvements in MEG and EEG source localization algorithms, one should compare the connectivity analysis between sensor and space to be aware of the spatial filtering effect. Field spread effect can never be totally diminished or abolished after applying a source localization technique (Schoffelen and Gross, 2009); however, applying source connectivity will further enhance both the results of the present study and the interpretation of active areas related to the tasks by taking the advantage of a larger number of fMRI arithmetic studies.

## Conclusion

In the present study, we attempted to provide the first demonstration of dynamic orchestration between θ oscillations and modality-specific high α_2_ oscillations as the link between central executive system (F^θ^) and storage buffer functions (PO^α^_2_) in a WM-oriented multilevel mental task. Our analysis focused on the comparison between correct and wrong answers to reveal significant differences between and within WM subsystems. To uncover the distinct roles of amplitude and phase under the notion of connectivity, we adopted a large repertoire of connectivity estimators including our novel approach, which is one of the first techniques that can uncover the strength, direction, and lag between different frequencies. We successfully discriminated correct and wrong answers based on FCGs tabulating the interactions between F^θ^ and PO^α^_2_ estimated with the novel dSTE. Zero time-lag between bilateral F^θ^ and right PO^α^_2_ could also indicate mental calculation performance independently of task difficulty. Overall, our results highlight the significant role of integrated activity between F^θ^ and PO^α^_2_ via the strength of their causal interactions and the precise timing of their coordination for arithmetic performance.

## Author Contributions

Conception of the research: SD. Methods design and data analysis: SD. Drafting the manuscript: SD. Critical revision of the manuscript: YS, NT, and AB.

## Conflict of Interest Statement

The authors declare that the research was conducted in the absence of any commercial or financial relationships that could be construed as a potential conflict of interest.

## Footnotes

1 We detected the peaks of θ (6 Hz) cycles by estimating zero crossings from positive to negative values of the derivative of the time series recorded from the FZ sensor and filtered at 6 Hz. The same procedure was employed across trials, subjects, and conditions
