# Supplementary material for: Causal Interactions between Frontalθ – Parieto-Occipitalα2 Predict Performance on a Mental Arithmetic Task
Source: Front Hum Neurosci. 2016 Sep 14;10:454. doi: 10.3389/fnhum.2016.00454 (PMC5022172; doi:10.3389/fnhum.2016.00454)
Supplement: Supplementary file 1 [file Data_Sheet_1.DOCX]

Supplementary Material

Causal interactions between Frontal^θ^ – Parieto-Occipital^α2^ **predict performance** in a mental arithmetic task

Stavros I. Dimitriadis ^1, 2, 3, 4, *^, Yu Sun ^5^, , Nitish V. Thakor ^5^, Anastasios Bezerianos ^5^

^1^ Institute of Psychological Medicine and Clinical Neurosciences, Cardiff University School of Medicine, Cardiff, United Kingdom

^2^ Cardiff University Brain Research Imaging Center (CUBRIC), School of Psychology, Cardiff University, Cardiff, United Kingdom

^3^ Artificial Intelligence and Information Analysis Laboratory, Department of Informatics, Aristotle University, Thessaloniki, 54124, Greece

^4^ Neuroinformatics.GRoup, Department of Informatics, Aristotle University, Thessaloniki, Greece

/ http://neuroinformatics.gr/

^5^ Singapore Institute for Neurotechnology (SINAPSE), Centre for Life Sciences, National University of Singapore, 28 Medical Drive, 117456, Singapore

* Correspondence should be addressed:

S.I. Dimitriadis, Institute of Psychological Medicine and Clinical Neurosciences, Cardiff University, School of Medicine, Cardiff, United Kingdom

email: [DimitriadisS@cardiff.ac.uk](mailto:DimitriadisS@cardiff.ac.uk) , [stidimitriadis@gmail.com](mailto:stidimitriadis@gmail.com), Tel: +44-02920-876506

**Contents:**

**Section 1. Power Spectrum Analysis (PSA) ……………………………………………………..4**

**Section 2. Different types of Phase Synchronization …………………………………………...4**

**Section 3. Delay Symbolic Transfer Entropy (dSTE) and Significant Test …………………..7**

**Section 4. Time Lag Estimation …………………………………………………………………11**

**Section 5. Directed PLI (dPLI) ………………………………………………………………….12**

**Section 6. Phase-to-amplitude (PAC) Cross-Frequency Coupling…………………………….13**

**Section 7. Experimental Validation ……………………………………………………………..15**

**Section 8. Reaction Time and Accuracy…………………………………………………………16**

**References …………………………………………………………………………………………17**

**Section 1. Power Spectrum Analysis (PSA)**

We converted the EEG signal per sensor from the time domain into the frequency domain (Welch periodogram; matlab R2012a; window

=

128 samples; overlap

=

64 samples; nfft

=

128; sample frequency

=

256

Hz). Per sensor, the absolute power spectrum was estimated within θ:5 – 6 Hz and α_2_ : 10 – 13 Ηz  where the frequency bins were 3 and 7 correspondingly.

**Section 2. Different types of Phase Synchronization**

1. **Within and Cross-Frequency Phase Synchronization**

PLV computation is based on estimates of instantaneous phase obtained from the convolution of *Morlet* wavelets with the EEG signals $x_{i}\left(n\right)$ filtered within one of the frequency bands under study. The resulting *Dyadic Wavelet Transform* of a discrete sequence $x\left(n\right)$ sampled with time spacing $\delta_{t}$ and consisting of $N$ data points ($n=0,1,\ldots,N-1$) is denoted as:

$$W^{x(n,s)}=\sqrt{\delta_{t}/s}\sum_{n'=0}^{N-1} x\left(n'\right)\psi_{0}^{*}\left(\left(n'-n\right)\delta_{t}/s\right)\text{ (A.1)}$$

where ‘*’ denotes complex conjugation with the consecutive scaled and translated versions of the principal wavelet function, the complex Morlet wavelet $\psi_{0}\left(n\right)$:

$$\psi_{0}\left(n\right)=\pi^{-1/4}e^{iw_{0}n}e^{-n^{2}/2}\text{ (A.2)}$$

where $\omega_{0}$ is the nondimensional frequency, here taken to be 6 ([Torrence and Compo, 1998](#_ENREF_64)). A set of different scales $s$ is implied in eq. (A.1). Writing the scales as fractional powers of two yields the following:

$$s_{j}=s_{0}2^{j\delta_{j}},\text{ }j=0,1,\ldots,J\text{ }\left(J=\delta_{j}^{-1}\text{log}_{2}\left(N\delta_{t}/s_{0}\right)\right)\text{ (A.3)}$$

where $s_{0}$ is the smallest resolvable scale and $J$ the largest scale; our analysis starts by estimating the optimal $\delta_{j}$ for each band/condition.

The instantaneous phase $\psi^{X_{i}}\left(n,f\right)$ is then calculated as follows:

The successive phase values (originally ranging in [-π, π]) underwent an unwarping transform to get rid off discontinuities (in accordance with the phase correction algorithm described in (Freeman and Rogers, 2002). $s_{1}/s_{2}$ refers to the scale limits, $\Delta$ denotes the corresponding range and $f$ the studying frequency band ([Lachaux et al. , 2000](#_ENREF_35)).

The phases were calculated by wavelet transforms using Morlet’s wavelets with a Gaussian shape in the time domain (standard deviation σ_t_) and with the frequency domain (standard deviation σ_f_) located around the center frequency (

f

) ([Tallon Baudry et al., 1997](file:///I:\SINGAPORE-PROJECTS\cognitive-workload\manuscripts\methods-cognitive-levels\A%20tensorial%20approach%20to%20classify%20working%20memory%20load%20related%20to%20mental%20tasks%20from%20EEG%20functional%20connectivity%20graphs%5b5%5d.docx#_ENREF_41); [Torrence and Compo, 1998](file:///I:\SINGAPORE-PROJECTS\cognitive-workload\manuscripts\methods-cognitive-levels\A%20tensorial%20approach%20to%20classify%20working%20memory%20load%20related%20to%20mental%20tasks%20from%20EEG%20functional%20connectivity%20graphs%5b5%5d.docx#_ENREF_44)).

To quantify the phase relations between any two electrodes, the Phase Locking Value (PLV) for each electrode pair and for the window that begins with the onset of the stimulus and ends with the last peak of theta (6 Hz) cycle[[1]](#footnote-1) was estimated based on Morlet’s Wavelets and was defined as:

where PLV quantifies the phase difference between every pair of electrodes $k$, $l$ within the window W^1^.

The within–frequency phase synchronization equation (A.5) was applied to estimate functional connectivity in Frontal (F) brain areas in θ band and in Parieto-occipital (PO) brain regions in α_2_ band (10 – 13 Hz). Our analysis focused on 25 out of 64 channels located in F and PO brain areas which are the following: FZ, FP1, AF3, F3, F7, FC5, FC1, FC6, FC2, F4, F8, FP2, AF4 PZ, P7, P8, P5, P6, PO7, PO8, PO3, O1, OZ, O2, PO4 (see Section 3.4.2).

We also calculated the phase-phase cross-frequency coupling (CFC) between the θ (6-Hz) and α_2_ (10 – 13 Hz) oscillations at both within electrode located over F and PO sites but also as a cross-talk coordinate mechanism between the two distinct functions of WM (storage buffer and central executive buffer) located in both sites. The PLV formula as applied as the phase difference between two θ phases (2×Φ_6 Ηz_) and the α_2_ phase (Φ_10 - 13 Ηz_) at the electrodes located in both F and PO sites but also between them. The following equation estimates the cross-talk between F^θ^ and PO^α2^

where k,l denote the sensors located over F and PO brain sites correspondingly.

The cross-talk of θ and α_2_ within F^θ: α2^ and PO^θ: α2^ is described with the following equation:

where k denotes the sensor located over F or PO brain sites.

1. **Accessing significant functional phase interactions**

It is common practice to trim the initial estimates of functional connectivity so as to null out insignificant couplings that always appear due to random fluctuations in the time series. Based on a Rayleigh test for the uniformity of PLV values, we calculated the significance of each value (significance is calculated as *p* = exp(*−N*_trials_$PLV_{AVE}^{2}$), where PLV_AVE_ is the average PLV value across trials; Fisher,1989). To correct for multiple testing, the false discovery rate (FDR) method was adopted ([Benjamini and Hochberg, 1995](file:///I:\SINGAPORE-PROJECTS\cognitive-workload\manuscripts\methods-cognitive-levels\A%20tensorial%20approach%20to%20classify%20working%20memory%20load%20related%20to%20mental%20tasks%20from%20EEG%20functional%20connectivity%20graphs%5b5%5d.docx#_ENREF_2)). A threshold of significance was set such that the expected fraction of false positives was restricted to *q ≤* 0.00001. The procedure was followed for each Cognitive Workload Level and subject independently ([Dimitriadis et al., 2012](file:///I:\SINGAPORE-PROJECTS\cognitive-workload\manuscripts\methods-cognitive-levels\A%20tensorial%20approach%20to%20classify%20working%20memory%20load%20related%20to%20mental%20tasks%20from%20EEG%20functional%20connectivity%20graphs%5b5%5d.docx#_ENREF_11)d).

**Section 3. Delay Symbolic Transfer Entropy (dSTE) and Significant Test**

**a) Delay Symbolic Transfer Entropy (dSTE)**

In principle, asymmetric dependences between coupled systems can be detected with measures that share some of the properties of mutual information (Shannon and Weaver,1949) and take into account the dynamics of information transport. Transfer entropy (Schreiber,2000), which is related to the concept of Granger causality (Granger, 1969), has been proposed to distinguish effectively driving and responding elements and to detect asymmetry in the interaction of subsystems. By appropriate conditioning of transition probabilities this quantity has been shown to be superior to the standard time delayed mutual information, which fails to distinguish information that is actually exchanged from shared information due to common history and input signals. Various techniques have been proposed to estimate transfer entropy from observed data. Most techniques, however, make great demands on the data, require fine-tuning of parameters, and are highly sensitive to noise contributions, which limits the use of transfer entropy to field applications (Verdes, 2005 ; Chavez et al., 2003).

A Symbolic Transfer Entropy (STE) was proposed to overcome the limitations of optimized parameters needed for the estimation of Transfer Entropy (Staniek and Lehnertz, 2008). In the present study, we adopted Neural Gas algorithm (Martinez et al., 1993) as an appropriate technique for create a common codebook for a multichannel dataset (Dimitriadis et al., 2012).

We describe here the algorithmic steps, with which we transcribe the temporal dynamics from any pair of sensors into two distinct symbolic timeseries that share a common codebook (set of symbols). The size and content of the codebook is data-dependent and estimated every time causal relationships are to be inferred from a pair of recorded signals. The associated computational burden is kept low thanks to the employed unsupervised algorithm (that is Neural-Gas – Dimitriadis et al., 2016a).

Given the signals ^A^x_t_ and ^B^x_t_ from a pair of channels A and B, time-delay vectors are first reconstructed from each time series. These vectors take the form $\mathbf{x}_{\mathbf{t}}=\{x_{t},x_{(t+\tau)},...,x_{(t+(m-1)\tau)}\}$ where $m$ is the embedding dimension, $\tau$ denotes the time lag and t=1,2,..,T runs over the time points.

Then, the two individual sequences of time-delay vectors, are collectively gathered in data matrices :

Next, the two trajectories are brought to a common reconstrcuted state space by forming the overall data matrix

${}^{AB}\mathbf{X}\text{ }_{\text{[2T}\times\text{m]}}=\text{ }[\text{ }{}^{\mathbf{A}}\mathbf{X}\text{ }|\text{ }{}^{B}\mathbf{X}\text{ }]\text{ }$ [A.8]

The partition of all the tabulated m-dimensional vectors into groups of homogenous patterns is the most direct way to summarize the temporal variations in the activations of these two subsystems and describe them with a common vocabulary.

In our approach, a codebook of $k$ code vectors is designed by applying the NG algorithm to the data matrix ^AB^**X** which is of size [~2T×m]. The NG algorithm is an artificial neural network model, which converges efficiently to a small number $k<<T$ of codebook vectors $\{\mathbf{M}_{\mathbf{i}}\}_{i=1:k}$ using a stochastic gradient descent procedure with a soft-max adaptation rule that minimizes the average distortion error ([Martinetz et al., 1993](#_ENREF_32)).

In the encoding stage, each of the 2$T$ vectors is assigned to the nearest code vector. By replacing the original vectors with the assigned code vectors, we can rebuild the two vectorial time series with a measurable error. If we denote the reconstructed (i.e. decoded) version of the vectorial timeseries as ${}^{AB}\mathbf{x}^{\mathbf{rec}}(t)$, we can estimate the fidelity of the overall encoding procedure with the following index ,which is the total distortion error divided by the total dispersion of the original vectors:

$n_{Distortion}=\frac{\sum_{t=1}^{2T} \left\|^{AB}\mathbf{x}(t)-{}^{AB}\mathbf{x}^{\mathbf{rec}}(t)\right\|^{2}}{\sum_{t=1}^{2T} \left\|{}^{AB}\mathbf{x}(t)-\overline{x}\right\|^{2}},\text{ }\overline{\mathbf{x}}=\frac{1}{2T}\sum_{t=1}^{2T} {}^{AB}\mathbf{x}(t)$ [A.9]

The smaller the $n_{Distortion}$, the better the encoding. This index gets smaller with the increase of $k$, while reaches a plateau for a relative small value of $k$. In the present study, we considered encoding to be acceptable if it was produced with the smallest $k$ that satisfied the condition that $n_{Distortion}$ should be less than 5%. Hence, we repeatedly applied the NG algorithm with increasing k and measured the reconstruction quality. In this way we defined the optimal $k_{0}$, which in turn defined the codebook to use in the subsequent symbolization scheme. At the vector-quantization stage, each vector of **^A^X** and **^B^X** is assigned (according to the nearest-prototype rule) to the most similar among the derived codevectors $\{\mathbf{M}_{\mathbf{i}}\}_{i=1:ko}$. This step completes the mapping of original timeseries to two symbolic time series ^A^s_t_ and ^B^s_t_, $t=1,2,\ldots T$, which in mathematical notation reads as follows:

[A.10]

In the derived symbolic time series, the temporal dynamics of a pair of neural subsystems are encoded as transitions among adaptively-defined (i.e. data-dependent) symbols.

We adopted Ragwitz criterion for optimizing the embedding dimension d and the embedding delay τ (Ragwitz and Kantz,2002). Optimality of the embedding refers to a minimal prediction error for future samples of the time series. The Ragwitz criterion predicts the future of a signal based on estimates of the probability densities of future values of its nearest neighbors after embedding. The adopted method is based on the minimization of mean squared prediction error (Ragwitz and Kantz,2002 ; Lindner et al., 2011).

b) **Quantifying Effective Connectivity**

Provided a pair of symbolic sequences ^A^s_t_ and ^B^s_t_, the relative frequency of symbols can be used to estimate joint and conditional probabilities, and to define symbolic transfer entropy (STE) as follows

$STE_{{}^{BA}}=\sum p({}^{A}s_{t+\delta},{}^{A}s_{t},{}^{B}s_{t})\log\frac{p({}^{A}s_{t+\delta}/{}^{A}s_{t},{}^{B}s_{t})}{p({}^{A}s_{t+\delta}/{}^{A}s_{t})}$ [A.11]

where the sum runs over all symbols and *δ* denotes a time step.

Effective connectivity is defined as “the influence one system exerts over another” (Granger, 1969; Ito et al., 2011). In the context of brain networks, effective connections are directed from one brain area to another. To account for the time-delay between brain activation signals from distant areas, we modified the previous definition :

$dSTE_{BA}=\text{ }STE_{BA}(d)=\sum p({}^{A}s_{t+1},{}^{A}s_{t},{}^{B}s_{t+1-d})\log\frac{p({}^{A}s_{t+1}/{}^{A}s_{t},{}^{B}s_{t+1-d})}{p({}^{A}s_{t+1}/{}^{A}s_{t})}$ [A.12]

where $d$ is the time delay between and the driving and the driven system. The log is with base 2, thus STE_BA_ is given in bits. STE_AB_ is defined in complete analogy. The directionality index  ΔdSTE_AB_=dSTE_AB_-dSTE_BA_  quantifies the preferred direction of information flow and gets positive values for unidirectional couplings with A as the driver and negative values for B driving A. For symmetric bidirectional couplings ΔdSTE is approximately zero. The formulation of TE with a time delay was first proven to be correct in a recent study, which presented a robust method for neuronal interaction delays (Wibral et al., 2013).

To detect significant causal interactions between two brain regions (considered as subsystems A and B), we adopted a well-known technique described in ([Chavez et al., 2003](#_ENREF_8); [Verdes, 2005](#_ENREF_54) ; [Lizier et al., 2011](#_ENREF_30) ; Vicente et al., 2011). The original approach has been developed for TΕ but can easily be applied to its symbolic counterpart as well. The null hypothesis H_0_ of the test is that the state trantitions ${}^{\text{A}}\mathbf{x}_{\text{n}}\to{}^{\text{A}}\mathbf{x}_{\text{n}+1}$ of the destination system A have no temporal dependence on the states of the source system B. We form a distribution of dSTE measurements $\{{}^{Ho}dSTE_{BA}\}_{r=1:1000}$ under this condition by repeatedly applying the following algorithmic steps:

Step_i: Generate a surrogate time-series by permuting the elements of the source symbolic time series ^B^s_t_ ;

Step_ii: Estimate an instantiation of the ''randomized'' dSTE_BA_ using the ^A^s_t_ and the surrogate ^B^x_t_ in eq.5.

We can then determine an one-sided *p*-value that corresponds to the likelihood that the actual observed value, namely observed-dSTE_BA_, is within the range of values of the distribution $\{{}^{Ho}dSTE_{BA}\}_{r=1:1000}$. This can be done by directly estimating the proportion of ''randomized'' dSTE_BA_ that are higher than the observed-dSTE_BA_ value ([Lizier et al., 2011](#_ENREF_30) ; [Dimitriadis et al., 2012](#_ENREF_15)).

**Section 4. Time Lag Estimation**

To reinforce our results related to time lag estimation, we adopted a second approach apart from dSTE which gives information for the strength, the direction and the time lag of causal interactions (Adhikari et al., 2010)**.** After the instantaneous amplitude for all the points for each pair of signals from Frontal^θ^  and Parieto-Occipital^α2^ was calculated, the cross-correlation between the amplitudes of the two signals was computed with the MATLAB function xcorr, over lags ranging from +50%*trial^length^ to −50%*trial^length^ s (where trial^length^ denotes the length of trial in sec). The mean amplitude was first subtracted from each vector prior to cross-correlating them, as the DC component of a signal has no relevance for a cross-correlation. The lag at which the cross-correlation peaked was then determined. The significance of each Frontal^θ^  and Parieto-Occipital^α2^ amplitude cross-correlation was verified before inclusion in additional analyses using a bootstrap procedure. Frontal^θ^  and Parieto-Occipital^α2^ amplitude envelopes were randomly shifted two θ cycles relative to each other 1000 times. The shifted amplitude envelopes were then cross-correlated, yielding a distribution of cross-correlation peaks expected by chance. The original cross-correlation was considered significant if its peak value was greater than 95% of these randomly generated cross-correlation peaks.

**Section 5. Directed PLI (dPLI)**

For the estimation of causal relationships between phases of different frequencies, we adopted the directed Phase Lag Index (dPLI;Stam et al., 2012). Τhe dPLI gets the phase difference between two time series A and B where there are three cased :

1. Most of the phase differences between two time series are in the interval of $0<=\Phi(t)<\pi$ and signal A is consistently leading in phase domain signal B with a dPLI > 0.5
2. Phase difference of the two signals are on average π radians out of phase where we cannot say anything about who drives who and
3. Most of the phase differences between two time series are in the interval of $-\pi<=\Phi(t)<0$ and signal B is consistently leading in phase domain signal A with a dPLI < 0.5

In the present study to access only the significant causal phase relationships between every pair of sensors across Frontal^θ^  and Parieto-Occipital^α2^ , determined via surrogate data (Theiler et al., 1992). One thousand surrogate time-series were generated by cutting at single point at a random location and exchanging the two resulting time courses (Canolty et al., ; 2006; Aru et al., 2014 ). Repeating this procedure leads to a set of surrogates with a minimal distortion of the original phase dynamics while destroying less the non-stationarity of the brain activity compared to shuffling the time series or cutting and rebuilding it in more than one time points. This procedure assures that the real and surrogate indices both have the same statistical properties. For each data set the surrogate dPLI (^s^dPLI) was then computed. We then determined an one-sided *p*-value for each PAC value that corresponded to the likelihood that the observed value could belong to the surrogate distribution. This was done by directly estimating the proportion of ''surrogate'' dPLI^s^ that were higher than the observed dPLI (Theiler et al., 1992). The p-value reflected the statistical significance of the observed dPLI-level (a very low value revealed that it could not have appeared from processes with no dPLI coupling).

To FDR method (Benjamini and Hochberg, 1995) was employed to control for multiple comparisons across all possible pairs of sensors between Frontal^θ^  and Parieto-Occipital^α2^  with the expected fraction of false positives set to
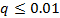
. Finally, we estimated the strength of the significant pair of dPLI across Frontal^θ^  and Parieto-Occipital^α2^ for each trial, CWL (5), condition (correct and wrong trials) and subjects. It is important to mention here that no significant causal relationship was detected with the Parieto-Occipital^α2^  as the driven area and so all the detected significant dPLI values were > 0.5 with the Frontal^θ^  leading Parieto-Occipital^α2^ .

**Section 6. Phase-to-amplitude (PAC) Cross-Frequency Coupling**

CFC estimates the strength of pairwise interactions and identifies the prominent interacting pair of frequencies, both between and within sensors (Canolty and Knight, 2010; Buzsaki, 2010; Buzsaki et al., 2013). Among available CFC descriptors, phase-amplitude coupling (PAC), which relies on phase coherence, is the one most commonly encountered in research (Cohen, 2008; Voytec et al., 2010). The PAC algorithm as adapted to continuous MEG multichannel recordings is described below.

Τhe more general case of CFC (i.e., between-sensor coupling) is described here—within-sensor CFC is derived by collapsing the two sensor indices to a common index. Let x(i_sensor_, t), be the MEG activity recorder at the i_sensor_-th site, and t=1, 2,.... T the successive time points. Given two frequency-limited signals x(i_sensor_,t) and x(j_sensor_,t), cross-frequency coupling is estimated by allowing the phase of the lower frequency (LF) oscillations to modulate the amplitude of the higher frequency (HF) oscillations. The complex analytic representations of each signal z_LF_(t) and z_HF_(t) are derived via the Hilbert transform (HT[.]).

$$\text{z}_{\text{LF}}\text{(t)}=\text{HT[x}_{\text{LF}}\text{(t)]}=\left|\text{z}_{\text{LF}}\text{(t)}\right|\text{ e}^{\text{i }\varphi_{\text{LF}}(t)}=A_{\text{LF}}\text{(t) e}^{\text{i }\varphi_{\text{LF}}(t)},\text{ z}_{\text{HF}}\text{(t)}=\text{HT[x}_{\text{HF}}\text{(t)]}=\left|\text{z}_{\text{HF}}\text{(t)}\right|\text{ e}^{\text{i }\varphi_{\text{HF}}(t)}=A_{\text{HF}}\text{(t) e}^{\text{i }\varphi_{\text{HF}}(t)}$$

Next, the envelope of the higher-frequency oscillations A_HF_(t) is bandpass-filtered within the range of LF oscillations and the resulting signal is submitted to an additional Hilbert transform to derive its phase dynamics component φ'(t)

$$\text{z'(t)}=\text{HT[ }A_{\text{HF,LF}}\text{(t) ]}=\text{ }\left|\text{z'(t)}\right|\text{ e}^{\text{i }\varphi'_{\text{HF}}(t)}=\left|\text{z'(t)}\right|\text{ e}^{\text{i }\varphi_{\text{LF}\to\text{HF}}(t)}\text{ }$$

which expresses the modulation of the amplitude of HF-oscillations by the phase of the LF-oscillations. Phase consistency between the two timeseries was measured by means of the original definition (Lachaux et al., 1999), as a synchronization index to quantify the strength of PAC.

The original PLV is defined as follows:

$$PLV=\frac{1}{T}*\sum_{t=1}^{T} e^{i(\varphi_{LF}(t) - \varphi_{HF}(t))}) (1)$$

For every sensor-pair, and between every pair of sensors across Frontal^θ^  and Parieto-Occipital^α2^ , we tested the null hypothesis H_0_: ''the observed PAC value comes from the same distribution as the distribution of surrogate PAC-values''. One thousand surrogate time-series were generated by cutting at single point at a random location and exchanging the two resulting time courses (Canolty et al., ; 2006; Aru et al., 2014 ). Repeating this procedure leads to a set of surrogates with a minimal distortion of the original phase dynamics while destroying less the non-stationarity of the brain activity compared to shuffling the time series or cutting and rebuilding it in more than one time points. This procedure assures that the real and surrogate indices both have the same statistical properties. For each data set the surrogate PAC (^s^PAC) was then computed. We then determined an one-sided *p*-value for each PAC value that corresponded to the likelihood that the observed value could belong to the surrogate distribution. This was done by directly estimating the proportion of ''surrogate'' PAC^s^ that were higher than the observed PAC (Theiler et al., 1992). The p-value reflected the statistical significance of the observed PAC-level (a very low value revealed that it could not have appeared from processes with no PAC coupling).

To FDR method (Benjamini and Hochberg, 1995) was employed to control for multiple comparisons across all possible pairs of sensors between Frontal^θ^  and Parieto-Occipital^α2^  with the expected fraction of false positives set to
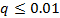
. Finally, we estimated the strength of the significant pair of PAC across Frontal^θ^  and Parieto-Occipital^α2^ for each trial, CWL (5), condition (correct and wrong trials) and subjects.

**Section 7. Experimental Validation**

**7.1 Trimmed FCGs**

We reduced the input for tensor analysis from FCGs of dimension (where denotes the original number of sensors) to sub-graphs of size $N'\times N'$ (with N’ equal the number of sensors extended spatially in bilateral F, in bilateral PO or in both brain areas ; see section 3.6 on the main manuscript). Each connection can be seen as feature used in TSA.

**7.2 Machine learning validation**

Incorporating both reaction times and Power Spectrum (θ, α_2_, θ/α_2_; Grimes et al., 2008 ; see Section 1 in the sup.material for the estimation of Power Spectrum) into a two-class classification scheme (correct vs wrong), we quantified the classification performance based on univariate data. We first normalized each feature independently to the range [0, 1]. In this study, we employed Laplacian score as a feature extraction technique for power spectrum and reaction time measurements ([He et al., 2005](#_ENREF_42)) and a k-NN classifier served as the predictor of correct versus wrong trials for each CWL. For the binary classification (correct vs wrong) for each of the five CWLs, we adopted a k-nearest neighbour classifier (k-NN) and a 10-fold cross-validation (CV) scheme.  To apply a statistical threshold to the corresponding laplacian score of each feature, we followed a bootstrapping randomized procedure where we shuffled the labels of correct – wrong trials 1.000 times and we reestimated the related laplacian scores for each of the selected features. After this randomization process, we estimated the mean + 2 st.d. (standard deviation) of the bootstrapped laplacian values as a statistical threshold for extracting the most informative features from response times and signal power. Our analysis was followed for each subject independently and the group-average classification performance was estimated. Laplacian score detected only signal power as informative features that can improve the binary classification of correct versus wrong trials at each CWL.

The TSA algorithm, followed by a k-nearest-neighbour classifier (k_nn_; with k=20), was tested on trial-based connectivity data from all the subjects independently for each CWL. The following results have been obtained through a cross-validation scheme that shuffle the trials and get 90% for training and 10% for testing. The cross-validation scheme was repeated 100 times and finally we estimated the mean and standard deviation across this high number of iterations. The selected options for TSA were: *Weight mode* = Ηeat Kernel; *Neighbour mode* = 10; Supervised learning; *Number of dimensions* = 6. The selection of the K_nn_ , neighbour mode and number of dimensions was driven by the maximization of the mean classification performance over the entire set of subjects (data not show here). The mean classification performance of TSA + k-NN applied to classify correct versus wrong trials independently for the five Cognitive Workload Levels in the entire set of 16 subjects.

**Section 8. Reaction Time and Accuracy**

S1 demonstrates the group-averaged response times (a) and the group-averaged accuracy scores for each CWL. The length of trials vary from 0.22 sec up to 7.51 sec in time and from 53 up to 1896 samples with a sampling frequency of 256 Hz. As we also mentioned in the main text (Section 2.6) , the total number of wrong trials within the group is 277 and distributed in each CWL as followed: Lv1 = 42, Lv2 = 53, Lv3 = 59, Lv4 = 62, and Lv5 = 61 (Lv = Level of Difficulty).

**
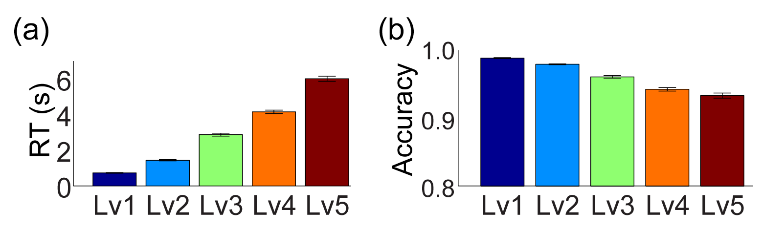
**

**S1**. Response times (a) and Accuracy scores (b) as a function of CWL.

**References:**

Adhikari A, Sigurdsson T, Topiwala MA, Gordon JA. (2010): Cross-correlation of instantaneous amplitudes of field potential oscillations: a straightforward method to estimate the directionality and lag between brain areas. J. Neurosci. Methods 191(2):191-200.

Aru, J., Aru, J., Priesemann, V., Wibral, M., Lana, L., Pipa, G., Singer, W., and Vicente, R. (2014) Untangling cross-frequency coupling in neuroscience. arXiv, arXiv:1405.7965, http://arxiv.org/abs/1405.7965.

Benjamini Y, Hochberg Y. (1995): Controlling the False Discovery Rate - a Practical and Powerful Approach to Multiple Testing. J. R. Stat. Soc. Ser. B-Stat. Methodol. 57(1):289-300.

Buzsáki, G and Watson B.O.(2012). Brain rhythms and neural syntax: implications for efficient coding of cognitive content and neuropsychiatric disease. Dialogues Clin. Neurosci. 14, 345–367

Buzsáki, G., Logothetis, N. and Singer, W.(2013). Scaling brain size, keeping timing: evolutionary preservation of brain rhythms. Neuron. 80,751-64.

Canolty, R.T. and Knight, R.T. (2010). The functional role of cross-frequency coupling, Trends Cogn. Sci. 14, 506-15.

Chavez M, Martinerie J, Le Van Quyen M. (2003): Statistical assessment of nonlinear causality: application to epileptic EEG signals. J. Neurosci. Methods 124(2):113-28.

Dimitriadis, S.I., Kanatsouli, K., Laskaris, N.A., Tsirka, V., Vourkas, M., Micheloyannis, S., 2012. A novel symbolization scheme for multichannel recordings with emphasis on phase information and its application to differentiate EEG activity from different mental tasks. Cogn Neurodyn. 2012 Feb; 6(1): 107–113.

Dimitriadis S, Sun Y, Laskaris N, Thakor N, Bezerianos A (2016a) Revealing cross-frequency causal interactions during a mental arithmetic task through symbolic transfer entropy: a novel vector-quantization approach. IEEE Trans.Neural Syst.Rehabil Eng [Epub ahead of print].

C. W. Granger, “Investigating causal relations by econometric models and cross-spectral methods,” *Econometrica*, pp. 424-438, 1969.

S. Ito, M. E. Hansen, R. Heiland *et al.*, “Extending transfer entropy improves identification of effective connectivity in a spiking cortical network model,” *PLoS One,* vol. 6, no. 11, pp. e27431, 2011.

Lachaux JP, Rodriguez E, Van Quyen ML, Lutz A, Martinerie J, Varela FJ. (2000): Studying single-trials of phase synchronous activity in the brain. International Journal of Bifurcation and Chaos 10(10):2429-2439.

M. Lindner, R. Vicente, V. Priesemann *et al.*, “TRENTOOL: a Matlab open source toolbox to analyse information flow in time series data with transfer entropy,” *BMC Neurosci.,* vol. 12, pp. 119, 2011.

J. T. Lizier, J. Heinzle, A. Horstmann *et al.*, “Multivariate information-theoretic measures reveal directed information structure and task relevant changes in fMRI connectivity,” *J. Comput. Neurosci.,* vol. 30, no. 1, pp. 85-107, Feb, 2011.

Martinetz TM, Berkovich SG, Schulten KJ. (1993): Neural-Gas Network for Vector Quantization and Its Application to Time-Series Prediction. IEEE Trans. Neural Networ. 4(4):558-569.

M. Ragwitz, and H. Kantz, “Markov models from data by simple nonlinear time series predictors in delay embedding spaces,” *Phys. Rev. E,* vol. 65, no. 5 Pt 2, pp. 056201, May, 2002.

TallonBaudry C, Bertrand O, Delpuech C, Pernier J. (1997): Oscillatory gamma-band (30-70 Hz) activity induced by a visual search task in humans. J. Neurosci. 17(2):722-734.

Stam, CJ and van Straaten, ECW. Go with the flow:Use of a directed phase lag index (dPLI) to characterize patterns of phase relations in a large-scale model of brain dynamics. Neuroimage Volume 62,3 1415-1428.

Torrence C, Compo GP. (1998): A practical guide to wavelet analysis. Bull. Amer. Meteorol. Soc. 79(1):61-78.

Verdes PF. (2005): Assessing causality from multivariate time series. Phys. Rev. E 72(2 Pt 2):026222.

M. Wibral, N. Pampu, V. Priesemann *et al.*, “Measuring information-transfer delays,” *PLoS One,* vol. 8, no. 2, pp. e55809, 2013.

1. The peaks of θ (6 Hz) cycles was detected by estimating zero crossings from positive to negative values of the derivative of the time series located in frontal brain areas filtered at 6 Hz. We used FZ sensor as a consistent indicator for last θ cycle across trials, subjects and conditions. [↑](#footnote-ref-1)
